# Current clinical applications of spectral tissue Doppler echocardiography (E/E' ratio) as a noninvasive surrogate for left ventricular diastolic pressures in the diagnosis of heart failure with preserved left ventricular systolic function

**DOI:** 10.1186/1476-7120-5-16

**Published:** 2007-03-26

**Authors:** Stephane Arques, Emmanuel Roux, Roger Luccioni

**Affiliations:** 1 Department of Cardiology, Aubagne Hospital, Aubagne, France; 2 La Timone University of Medicine, Marseille, France

## Abstract

Congestive heart failure with preserved left ventricular systolic function has emerged as a growing epidemic medical syndrome in developed countries, which is characterized by high morbidity and mortality rates. Rapid and accurate diagnosis of this condition is essential for optimizing the therapeutic management. The diagnosis of congestive heart failure is challenging in patients presenting without obvious left ventricular systolic dysfunction and additional diagnostic information is most commonly required in this setting. Comprehensive Doppler echocardiography is the single most useful diagnostic test recommended by the ESC and ACC/AHA guidelines for assessing left ventricular ejection fraction and cardiac abnormalities in patients with suspected congestive heart failure, and non-invasively determined basal or exercise-induced pulmonary capillary hypertension is likely to become a hallmark of congestive heart failure in symptomatic patients with preserved left ventricular systolic function. The present review will focus on the current clinical applications of spectral tissue Doppler echocardiography used as a reliable noninvasive surrogate for left ventricular diastolic pressures at rest as well as during exercise in the diagnosis of heart failure with preserved left ventricular systolic function. Chronic congestive heart failure, a disease of exercise, and acute heart failure syndromes are characterized by specific pathophysiologic and diagnostic issues, and these two clinical presentations will be discussed separately.

## Background

Congestive heart failure (HF) has become a highly prevalent medical syndrome in developed countries, which primarily affects older patients with a history of hypertension, coronary artery disease and diabetes mellitus [1]. There is now convincing evidence that nearly half of patients carrying a diagnosis of congestive HF do not have significant left ventricular (LV) systolic dysfunction and are referred as to suffering from HF with preserved LV systolic function (HFPSF) [2,3]. HFPSF is primarily defined as a clinical syndrome, however exercise intolerance, acute dyspnea and signs of fluid retention are nonspecific and obviously insufficient for definitely establishing the diagnosis [4-6]. The European Study Group has first proposed specific recommendations for the determination of LV diastolic dysfunction by invasive and noninvasive means as a diagnostic complement to the clinical syndrome in this setting [7], however, the diagnostic relevance of these additional criteria has been questioned recently [8]. Despite the absence of a large consensus [9-13], the analysis of mitral filling is currently regarded as an invaluable noninvasive tool for grading diastolic function in patients with suspected HFPSF [2]. A restrictive mitral filling pattern at Doppler echocardiography, which defines severe diastolic dysfunction and critical increase in LV filling pressures, readily identifies patients for whom symptoms are at least partly related to cardiac disease, however such a condition has been reported to account for approximately 10% of patients with HFPSF in a large, prospective population-based study [14]. Additional precision regarding LV diastolic pressures is required for the other patients to assess the severity of myocardial dysfunction and its contribution to symptoms [2]. Evidence of basal or exercise-induced pulmonary capillary hypertension is a hallmark of congestive HF at any level of functional disability regardless of LV systolic function [9-11], and such a marker of the severity of structural heart disease is attractive in the setting of HFPSF. Comprehensive Doppler echocardiography is currently recommended by the ESC and ACC/AHA guidelines as the single most useful diagnostic test for assessing LV ejection fraction and cardiac abnormalities in patients referred for suspected congestive HF, and non-invasively determined pulmonary capillary hypertension at rest or during exercise is likely to become a hallmark for the diagnosis of HFPSF. Our purpose is to review the current clinical applications of tissue Doppler echocardiography as a noninvasive surrogate for pulmonary capillary pressure in the diagnosis of HFPSF. Acute HF syndromes and chronic congestive HF, a disease of exercise, are characterized by specific diagnostic and prognostic issues [9-11], and these 2 clinical presentations will be discussed separately.

### Link between pulmonary capillary hypertension and the clinical syndrome of congestive HF: insights from hemodynamic evidence

During exercise, pulmonary capillary pressure remains unchanged, decreases or slightly increases in healthy subjects [15,16], though abnormal values >15 mmHg are occasionally observed for severely high degrees of exercise and cardiac output [17]. In physiological conditions, the absence of significant increase in LV diastolic pressures during exercise is partly due to cardiac and systemic adaptive mechanisms, such as increase in LV filling rate and enhancement of LV relaxation [18]. On the contrary, significant myocardial dysfunction is most commonly associated with basal or stress-induced left atrial hypertension as a consequence of maladaptive LV diastolic dynamics [9-11,15,16,19,20].

According to the Starling's principles regarding fluid exchanges through the capillary wall, the fluid movement across the pulmonary capillary membrane is governed by the integrity of the capillary membrane and a balance between opposing filtrative and absorbing forces:

Fluid movement = k(CHP - IHP) + k(πif - πc)

Where k = filtration coefficient defining the fluid conductance across the capillary membrane, CHP = intra-capillary hydrostatic pressure, IHP = interstitial hydrostatic pressure, πif = colloid osmotic pressure of interstitial fluid and πc = plasma colloid osmotic pressure.

Landmark studies have demonstrated experimentally that pulmonary capillary hypertension is the straightforward hemodynamic condition of cardiac pulmonary edema [21-23], along with the failure of lung fluid clearance capacities to rapidly compensate the excess of fluid transudation across the capillary membrane [24,25]. Total pulmonary extravascular water volume is closely related to the extent and the duration of increase in pulmonary capillary pressure [21-23]. However, low plasma colloid osmotic pressure due to hypoproteinemia is likely to modulate the pulmonary capillary pressure threshold of pulmonary edema formation, a condition that may be encountered in ICU's and elderly patients with multiple co-morbidities [26-28]. This pathophysiologic model is ascribed to acute HF syndromes but it can also be applied to the setting of exercise intolerance. Reduced exercise capacity has been found to be associated with basal or stress-induced pulmonary capillary hypertension in chronic congestive HF, irrespective of LV systolic function [16,29-31]. Sub-clinical interstitial pulmonary edema related to basal or exercise-induced pulmonary capillary hypertension is most likely to account for the reversible component of the failure of the alveolar-capillary interface [32-35], beside irreversible structural alterations such as extracellular matrix thickening and hypertrophy of the microvasculature [36]. Particularly, in a recent clinical study [35], Agricola et al have clearly established by noninvasive means the link between rest and peak-stress pulmonary capillary hypertension and basal and stress-induced interstitial pulmonary edema. In their work, Agricola et al have used ultrasound lung comets images as a reliable marker of extravascular lung water [37]. Elevated LV diastolic pressures directly contribute to reduced exercise capacity in the setting of chronic HF, along with reduced cardiac output, lung dysfunction and peripheral factors such as skeletal muscle abnormalities, deregulation of peripheral neural activity and early lactate formation [9,16,38]. One can therefore state that basal or stress-induced pulmonary capillary hypertension plays as a link between structural heart disease and symptoms, and noninvasive evidence of this hemodynamic condition offers the ability to reinforce the diagnosis of congestive HF in patients presenting with symptoms suggestive of congestive HF and normal LV ejection fraction.

### Reliability of spectral tissue Doppler echocardiography as a noninvasive surrogate for LV diastolic pressures

**Figure 1:**
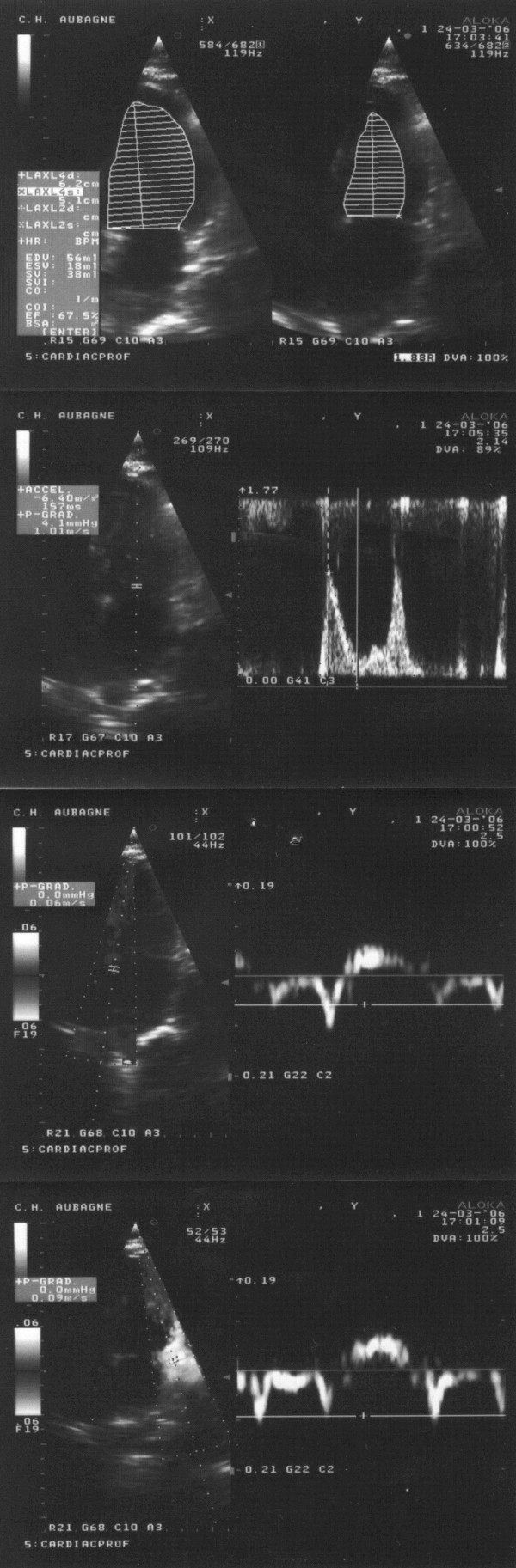
**Example of bedside measurement of spectral Doppler velocities (cm/s) in the apical 4-chamber view in a 82 years-old woman hospitalized for new-onset congestive heart failure with preserved left ventricular systolic function related to longstanding hypertension**. At presentation, the score of Boston criteria was 10 and B-type natriuretic peptide concentration 460 pg/ml. Bedside Doppler echocardiography performed before unloading therapy showed a left ventricular ejection fraction of 67% (upper part); peak E mitral velocity between the tips of mitral leaflets was 101 cm/s, spectral tissue Doppler peak early diastolic E' velocities at the septal (middle part) and lateral corner of mitral annulus (lower part) were 6 and 9 cm/s, respectively. The patient experienced a complete relief of symptoms and signs of pulmonary congestion under unloading therapy. Invasive left ventricular end-diastolic pressure recorded after clinical stabilization was 14 mm Hg.

Given the limitations inherent to invasive recordings, Doppler echocardiography has become an invaluable tool for non-invasively determining LV diastolic pressures routinely. Currently, the American Society of Echocardiography recommends the use of color M-mode and spectral tissue Doppler-derived indexes, E/Vp and E/E' respectively, for assessing left atrial pressure in clinical trials [39]. This is supported by a good compromise between easiness and reliability among a wide range of cardiac diseases. We will only discuss the clinical application of the E/E' ratio (see Figure 1) which has been consistently found to be more reproducible in daily practice [40].

The peak early diastolic mitral E velocity is primarily influenced by left atrial pressure, LV relaxation and LV systolic pressure in order of decreasing significance [41-43]. The spectral tissue Doppler-derived peak early diastolic E' velocity at mitral annulus is regarded as a noninvasive surrogate for LV relaxation, although its preload dependence has been reported in the setting of normal myocardial function [44-46]. The combination of E' with peak E velocity (i.e., E/E' ratio) is assumed to overcome the influence of ventricular relaxation on peak E velocity and reflect left atrial pressure, though a "pericardial constraint" effect may play a role in some patients with severely depressed LV systolic function and/or extremely high mean LV diastolic pressure [47].

Numerous clinical studies using spectral tissue Doppler for recording mitral annular velocities have produced convincing evidence of a positive, linear relation of E/E' with invasively determined mean LV diastolic pressure regardless of LV ejection fraction, rhythm and heart rate [45,48-60]. Some studies have also established that variations in E/E' accurately track changes in LV diastolic pressures under (un)loading therapy [48,55,56]. Clinical studies that found significant correlation between the spectral E/E' ratio and invasively determined pulmonary capillary wedge pressure or mean LV diastolic pressure in the setting of preserved LV systolic function are listed in Table 1. Tissue Doppler echocardiography is widely used for grading LV diastolic function in daily practice, particularly for differentiating the pseudonormal mitral filling pattern (which corresponds to moderate diastolic dysfunction and elevated filling pressures) from the normal filling (normal diastolic function and filling pressures) [14]. However, it appears of paramount importance to emphasize that some studies have produced convincing evidence that E/E' provides additional precision regarding LV filling pressures [49,52] and diagnosis (see next paragraph) beyond the mitral filling pattern. Some limitations should be considered before interpreting tissue Doppler results: 1) Color tissue Doppler and spectral tissue Doppler are not interchangeable [61]. 2) E' and E/E' are influenced by the mitral annular side of measurement and the level of LV ejection fraction [54]. 3) E/E' has been found to be more accurate than natriuretic peptides for non-invasively determining LV diastolic pressures [56]; nevertheless, E/E' allows only a semi-quantitative assessment and values of >10, >12 and >11 for lateral, septal, and average E/E', respectively, can be proposed for predicting pulmonary capillary pressure >15 mmHg in the presence of preserved LV systolic function [54]. 4) E/E' has been reported to be unreliable for predicting LV diastolic pressures in healthy subjects [62], organic mitral valve disease [63,64], and basal LV wall motion abnormalities related to left bundle branch block, paced-rhythm, myocardial infarction and cardiopulmonary bypass [65]. 5) E' and E/E' are influenced by age in healthy subjects [66-68]. Age-related increases in E/E' do not seem to be a consequence of higher LV diastolic pressures since left atrial pressure does not significantly rise in healthy elderly subjects [69,70]. Unexpected high values for E/E' are not uncommon in this setting, and underlying structural heart disease at comprehensive Doppler echocardiography should be taken into consideration before interpreting tissue Doppler echocardiography.

**Table 1:** Clinical studies that achieved a significant relation of spectral tissue Doppler E/E' ratio with invasive pulmonary capillary wedge pressure or mean left ventricular diastolic pressure in patients with preserved left ventricular systolic function

**Reference**	**Population study**	**Number of patients**	**r-value**	**LV ejection fraction**
Sundereswan (1998)^48^	Heart transplants	50	0.8 (L)	56 ± 12%
Nagueh (1998)^49^	ICU/Catheterism	49	0.72 (L)	> 45%
Nagueh (1999)^50^	HCM	35	0.76 (L)	> 50%
Sohn (1999)^51^	Atrial fibrillation	27	0.79 (S)	53 ± 11%
Ommen (2000)^45^	Catheterism	64	0.47 (S)	> 50%
Kim (2000)^52^	Catheterism	167	0.74 (S)	> 50%
Gonzalez (2002)^53^	ICU	32	0.54 (L)	> 50%
Rivas-Gotz (2003)^54^	ICU/Catheterism	55	0.7 (L)	> 50%
Bruch (2004)^57^	Aortic stenosis	23	0.75 (S)	59 ± 11%
Bruch (2005)^58^	Diastolic HF	28	0.56 (A)	> 45%
Hadano (2005)^59^	Catheterism	65	0.54 (L)	> 50%
Paelinck (2005)^60^	Chronic hypertension	18	0.85 (S)	58 ± 7%

Catheterism: clinically indicated catheterism; HCM: hypertrophic cardiomyopathy; HF: heart failure; ICU: intensive care unit; LV: left ventricular; (L): lateral E/E' ratio; (S): septal E/E' ratio; (A): average E/E' ratio.

### Usefulness of spectral tissue Doppler echocardiography in predicting HFPSF in the setting of exercise intolerance

Symptoms of dyspnea and fatigue usually characterize exercise intolerance in chronic congestive HF (stage C of the ACC/AHA classification for chronic HF) [9], regardless of LV ejection fraction [71]. Exercise intolerance correlates with maximal oxygen consumption but generally underestimate the impairment of functional capacity [72]. Preserved LV systolic function is reported to account for nearly half of patients carrying a diagnosis of congestive HF, however 3 recent clinical studies have questioned isolated diastolic dysfunction as a frequent cause of exertional dyspnea since alternative explanations, such as obesity and respiratory diseases, are found in most patients with normal LV ejection fraction [6,73,74]. A recent prospective study has confirmed the lack of specificity of the symptom of exertional dyspnea for the diagnosis of congestive HF (inconclusive positive predictive value of 48%) in a large, unselected patient group [75]. Non-invasively determined pulmonary capillary pressure at rest and during exercise is likely to be helpful for establishing the cardiac contribution to exercise intolerance, particularly among patients with confounding co-morbid conditions such as anemia, obesity and respiratory disease, which are frequently observed in this clinical setting [14].

Exercise-induced changes in the E/E' ratio are adequately recorded in most patients. In healthy subjects, E/E' remains unchanged during exercise because of a proportional increase in E and E' velocities [76]. It has been evidenced that E/E' accurately reflects changes in LV diastolic pressures during exercise [77,78]. Talreja et al have first reported the reliability of the septal E/E' ratio in reflecting variations of invasive LV diastolic pressures during exercise in 12 patients with exertional dyspnea and LV ejection fraction >50%; septal E/E' > 15 was given to be predictive of a pulmonary capillary pressure >20 mm Hg [77]. Confirmatory findings have been later reported by Burgess et al who recorded septal E/E' and invasive mean LV diastolic pressure at rest and during exercise in 37 patients with a mean LV ejection fraction of 58 ± 12%; the regression equation was similar for resting and exercise data, E/E' > 13 indicating a mean LV diastolic pressure >15 mm Hg [78]. Several works have successfully addressed the usefulness of the spectral tissue Doppler-derived E/E' ratio at rest, as well as during exercise, in predicting peak oxygen consumption used as a hallmark of functional disability in several clinical settings [78-86]: hypertrophic cardiomyopathy, chronic systolic HF, chronic LV systolic dysfunction, permanent atrial fibrillation, coronary artery disease with preserved LV systolic function and clinically indicated treadmill exercise (see Table 2). The weak relation of E/E' with maximal oxygen consumption that is observed in unselected patient populations emphasizes the multifactorial origin of exercise intolerance in congestive HF [87].

**Table 2:** Clinical studies that investigated the relation of the spectral tissue Doppler E/E' ratio with maximal exercise tolerance

**Reference**	**Population study**	**Number of patients**	**Relation with PVo2**	**Spectral tissue Doppler index measured at rest**
Matsumura^79^	HCM	85	-0.42* (p < 0.001)	Lateral E/E' ratio
McMahon^80^	HCM in children	80	-0.74* (p < 0.001)	Septal E/E' ratio
Ha^81^	HCM	29	-0.47* (p = 0.01)	Septal E/E' ratio
Smart^82^	Chronic systolic HF	95	-0.31* (p = 0.001)	Average E/E' ratio
Lee^83^	Permanent atrial fibrillation	73	-0.35^† ^(p = 0.03)	Septal E/E' ratio
Hadano^84^	Chronic LV systolic dysfunction	53	-0.68* (p < 0.001)	Lateral E/E' ratio
Van de Veire^85^	CAD and preserved LV systolic function	142	p = 0.005^‡^	Septal E/E' ratio
				
**Reference**	**Population study**	**Number of patients**	**Relation with METs**	**Spectral tissue Doppler index measured at rest**
Skaluba^86^	Clinically indicated treadmill exercise	121	-0.68* (p < 0.001)	Septal E/E' ratio
Burgess^78^	Clinically indicated treadmill exercise	166	-0.37* (p < 0.001)	Septal E/E' ratio
				
**Reference**	**Population study**	**Number of patients**	**Relation with METs**	**Post-exercise spectral tissue Doppler index**
Burgess^78^	Clinically indicated treadmill exercise	166	-0.44* (p < 0.001)	Septal E/E' ratio

CAD: coronary artery disease; HCM: hypertrophic cardiomyopathy; HF: heart failure; LV: left ventricular; METs: achieved metabolic equivalents; PVo2: peak oxygen consumption during exercise.

*: univariate regression analysis; ^†^: multivariate regression analysis; ^‡^: analysis of variance for E/E' between quartiles of PVo2.

**Figure 2:**
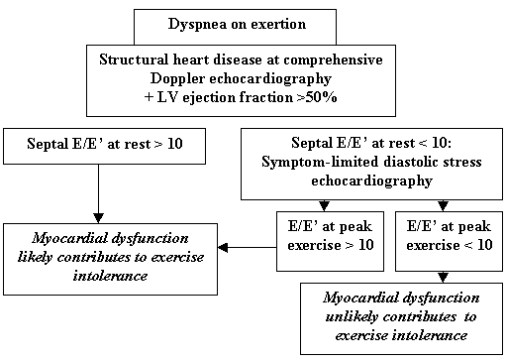
Proposition for a simplified diagnostic algorithm regarding the contribution of tissue Doppler echocardiography to the diagnosis of chronic heart failure with preserved left ventricular systolic function (stage C of the ACC/AHA classification) in patients presenting with exertional dyspnea, after excluding overt myocardial ischemia, significant valvular disease and arrhythmias; adapted from the references [9,67,76-78,88]. E: peak E mitral velocity; E': spectral tissue Doppler peak early diastolic velocity at the septal side of mitral annulus; LV: left ventricular. The use of a cut-off value of 13 would reinforce the positive predictive value but would lack the negative predictive value.

The clinical application of diastolic stress echocardiography (i.e., the recording of E/E' at rest and during a symptom-limited exercise) in the setting of exertional dyspnea is relevant for establishing the cardiac contribution to symptoms. In the study by Skaluba et al in which most patients presented with a normal LV ejection fraction, septal E/E' > 10 at rest was a strong, independent predictor of reduced exercise performance beyond LV systolic and diastolic function, as defined by the LV ejection fraction and the mitral filling pattern respectively [86]. In the study by Ha et al which focused on 45 patients with exertional dyspnea and normal LV ejection fraction, exercise duration was significantly less in patients with septal E/E' > 10 at rest or at peak exercise (7.1 ± 3.3 and 7.2 ± 2.5 minutes, respectively) compared to patients with septal E/E' < 10 both at rest and peak exercise (10.4 ± 3.7 minutes, p = 0.01) [88]. Confirmatory findings were reported by Burgess et al in a large patient population with preserved LV systolic function; particularly, exercise capacity was significantly less in patients with mild diastolic dysfunction and abnormal post-exercise septal E/E' (>10) compared with those with mild diastolic dysfunction but without abnormal post-exercise value for E/E' (7 ± 2.5 vs. 9.6 ± 2.6 METs respectively, p < 0.01) [78]. Septal E/E' > 10 measured at rest, at peak stress or immediately after exercise reinforces the likelihood that myocardial dysfunction (i.e., isolated diastolic dysfunction in the setting of normal LV ejection fraction and volumes) directly contributes to exertional symptoms, and may be very helpful for establishing the diagnosis of HFPSF (see Figure 2), along with other variables such as restrictive mitral filling pattern and increased natriuretic peptides concentrations [9,10,14]. Establishing underlying structural heart disease by comprehensive Doppler echocardiography is a necessary prerequisite before interpreting diastolic stress echocardiography (see Table 3), since values of 10–15 for septal E/E' are not uncommon in healthy elderly subjects [67]. The use of higher cut-off values (13 to 15) would reinforce the positive predictive value but would lack the negative predictive value of that test [78].

**Table 3:** Abnormalities at comprehensive Doppler echocardiography suggestive of structural heart disease without evidence of overt left ventricular systolic dysfunction and/or basal pulmonary capillary hypertension

2-dimensional and M-mode echocardiography*
-Mildly decreased left ventricular ejection fraction (45–54%) at 2D methods
-Abnormal left ventricular remodeling
-Left ventricular segmental wall motion abnormalities
-Increased left atrial size

**Doppler echocardiography**
-Doppler indexes suggestive of abnormal left ventricular relaxation: mitral inflow E/A ratio < 0.75; spectral tissue Doppler-derived lateral Ea velocity < 8 cm/s; color M-mode-derived Vp velocity < 45 cm/s
-Index of myocardial performance > 0.60
-Increased pulmonary artery pressure

*: for more information, report to the ASE Committee Recommendations. Lang RM, et al. *J Am Soc Echocardiogr *2005; 18: 1440–1463.

### Usefulness of bedside spectral tissue Doppler echocardiography in the emergency diagnosis of acute HFPSF

Acute, transient exacerbation of symptoms is a common clinical presentation of HFPSF, particularly among elderly patients [3]. The clinical diagnosis of acute HF syndromes is challenging in the emergency care setting [5]. In the landmark "Breathing not Properly Multinational" study, the Framingham score was reported to be 85% sensitive and 58% specific for the clinical diagnosis of congestive HF in a large, unselected patient population presenting with acute dyspnea [89]. Therefore, additional diagnostic methods are required in this clinical setting to accurately establish the diagnosis of acute congestive HF. The brain natriuretic peptide (BNP), a cardiac neurohormone that is secreted in response to myocardial stretch and volume overload, has been validated as a powerful and cost-effective diagnostic marker of congestive HF [90], and is extensively utilized as the first-line diagnostic complement to clinical and radiographic data in the acute care setting. A BNP level of < 100 pg/ml in sinus rhythm and < 200 pg/ml in arrhythmia is helpful for excluding the diagnosis of congestive HF with a likelihood of >90%; conversely, a BNP value of > 400–500 pg/ml readily identifies congestive HF with a positive predictive value of 90% [90,91]. Nevertheless, this biomarker is reported to be nondiagnostic at levels in the middle range (100–500 pg/ml) [90], and discrepant with the clinical judgment in a significant proportion of cases. Bedside Doppler echocardiography may ideally take place in the diagnostic strategy for such patients, particularly those with severe symptoms who experience a worse short-term outcome and require a rapid and accurate diagnosis for early improving the therapeutic care [90,92,93].

An echocardiographic LV ejection fraction of < 40–45% has been reported to be a powerful predictor of cardiac origin in patients presenting with acute dyspnea [89,94]. In the study by Logeart et al which focused on patients hospitalized for acute severe dyspnea, only 7 of the 82 patients with a bedside LV ejection fraction < 45% at presentation were classified having noncardiac cause of acute dyspnea at discharge [94]. In the "Breathing not Properly Multinational" study, LV ejection fraction was reported to have a positive predictive value of 88 and 94% at respective levels of ≤50% and ≤40% for the diagnosis of congestive HF in the setting of acute dyspnea [89].

**Figure 3:**
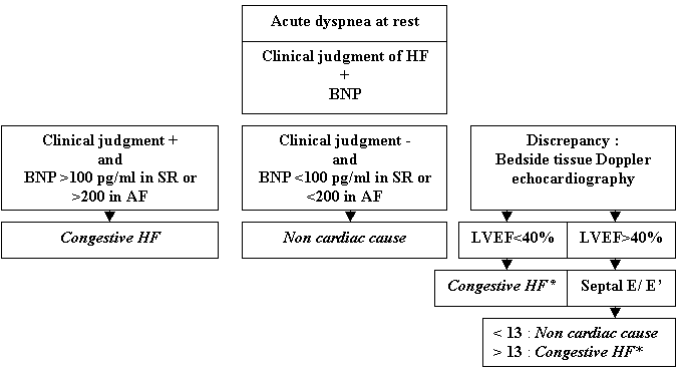
Proposition for a simplified diagnostic algorithm in the emergency diagnosis of acute congestive heart failure on admission, before tailored therapy, in patients presenting with acute dyspnea; adapted from references [5,89,91,94,98-102]. AF: atrial fibrillation; BNP: B-type natriuretic peptide; E: peak E mitral velocity; E': peak early diastolic velocity by spectral tissue Doppler at the septal side of mitral annulus; HF: heart failure; LVEF: left ventricular ejection fraction; SR: sinus rhythm. The clinical judgment of heart failure is based on the presence of prior history of heart failure and/or radiographic pulmonary edema. *: evidence of a restrictive mitral filling pattern reinforces the likelihood of acute congestive HF.

Noninvasive assessment of LV diastolic pressures at presentation, before tailored therapy, is clinically relevant particularly for patients with severe symptoms, inconclusive clinical, radiographic and biochemical data and preserved LV ejection fraction at echocardiography (see Table 4). It appears of paramount importance to emphasize that recordings after clinical stabilization may lead to inconclusive resting tissue Doppler results in patients for whom a positive response of pulmonary capillary hypertension has been reached under unloading therapy [95,96]. Dokainish et al have first reported the diagnostic accuracy of tissue Doppler echocardiography in a large patient population referred for suspected congestive HF with a wide range of symptoms (NYHA class II to IV) and LV ejection fractions [97]. The diagnostic accuracy of E/E' was similar to BNP regardless of LV ejection fraction; furthermore, these 2 methods were able to provide independent diagnostic information, supporting their complementary role in this setting. Three recent works have specifically addressed the usefulness of bedside tissue Doppler echocardiography as well as its incremental role over the clinical judgment and BNP testing in the emergency diagnosis of acute HFPSF in patients hospitalized for acute severe dyspnea; this noninvasive method was found to be accurate, even among patients with inconclusive BNP levels (100–400 pg/ml) or arrhythmia [98-100]. In these 3 studies, the clinical judgment of HF was based on the presence of prior history of congestive HF and/or radiographic evidence of pulmonary edema, 2 simple criteria that are among the most powerful predictors of congestive HF in the acute setting [5,101-103]; standard cut-off values for BNP in sinus rhythm (100 pg/ml) and permanent atrial fibrillation (200 pg/ml) were also utilized [91]. As expected, the agreement between the clinical judgment and BNP concentration achieved high accuracy for the etiologic diagnosis, and septal E/E' at a cut-off value of 13 correctly classified more than 85% of patients with discrepancy [98-100]. Septal E/E' at the cut-off value of 13 offers the ability to establish the diagnosis of congestive HF with a sensitivity of 76–82% and a specificity of 88–91% in the setting of acute dyspnea and preserved LV systolic function [98-100], however its combination with the clinical judgment and BNP testing (which plays as a marker of "chronic" filling pressures) may be more relevant to overcome the potential influence of low serum protein concentration in ICU's and elderly patients and to track false negatives due to transient increases in LV diastolic pressures (see Figure 3) [28,100]. Furthermore, along with tissue Doppler echocardiography, evidence of a restrictive mitral filling pattern reinforces the likelihood of acute congestive HF in this setting [94,99]. Bedside tissue Doppler echocardiography has been confirmed to be useful for the emergency diagnosis of acute HFPSF in patients with longstanding hypertension, achieving a better reproducibility and accuracy than the color M-mode Doppler-derived E/Vp ratio in this setting [104]. Another clinical study has provided confirmatory findings regarding the accuracy of tissue Doppler echocardiography for the diagnosis of acute congestive HF as well as its incremental role over inconclusive BNP levels in a small patient population with a wide range of LV ejection fractions [105].

**Table 4:** Clinical studies that investigated the accuracy of the spectral tissue Doppler E/E' for the diagnosis of acute congestive heart failure on admission before tailored therapy.

**Reference**	**Population study**	**Number of patients**	**Sensitivity-Specificity**	**Threshold value for E/E'**
Dokainish^97^	NYHA II to IV LVEF < 50%	76	92% – 72%	Average E/E' > 15
Dokainish^97^	NYHA II to IV LVEF > 50%	46	79% – 93%	Average E/E' > 15
Arques^98^	Acute dyspnea at rest LVEF > 45%	70	80% – 94.3% 76.7% – 91.4%	Average E/E' > 11.5 Septal E/E' > 13
Arques^99^	Acute dyspnea at rest LVEF > 50%, BNP 100–400 pg/ml	34	88.2% – 76.5% 76.5% – 88.2%	Average E/E' > 10.1 Septal E/E' > 13
Arques^100^	Acute dyspnea at rest, elderly LVEF > 50%, atrial fibrillation	41	81.8% – 89.5%	Septal E/E' > 13
Arques^104^	Acute dyspnea LVEF > 50%, hypertension	40	77.8% – 100%	Lateral E/E' > 11
Huang^105^	Acute dyspnea LVEF < 50%	-*	70.8% – 100%	Average E/E' > 16
Huang^105^	Acute dyspnea LVEF > 50%	-*	88.9% – 82.9%	Average E/E' > 11

BNP: B-type natriuretic peptide; LVEF: left ventricular ejection fraction;*: overall population of 92 patients.

## Conclusion

According to the ACC/AHA recommendations, the diagnosis of congestive HF is defined by the association of structural heart disease with symptoms compatible with the diagnosis. In the light of the present review, we can state that tissue Doppler evidence of pulmonary capillary hypertension at rest and unmasked during exercise is likely to become a hallmark for the diagnosis of congestive HF in the setting of preserved LV systolic function, regardless of the clinical presentation.

## Competing interests

financial competing interests

None

Non-financial competing interests

None
